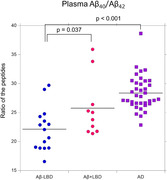# Plasma amyloid‐β40/42 ratio can distinguish co‐morbid Alzheimer’s disease pathology in patients with Lewy body disease

**DOI:** 10.1002/alz.084448

**Published:** 2025-01-09

**Authors:** Moeko Shinohara, Hidetomo Murakami, Yasuhiro Sakashita, Yukiko Mori, Junji Komatsu, Daiki Muramatsu, Sadao Hikishima, Kenjiro Ono

**Affiliations:** ^1^ Kanazawa University Graduate School of Medical Sciences, Kanazawa Japan; ^2^ School of Medicine, Showa University, Tokyo Japan; ^3^ Department of Neurology, Kanazawa University Graduate School of Medical Sciences, Kanazawa Japan

## Abstract

**Background:**

Although comorbidity with Alzheimer’s disease (AD) is not uncommon in Lewy body disease (LBD), no blood biomarkers which can identify AD pathology in LBD have ever been established.

**Objectives:**

To investigate if plasma amyloid‐β (Aβ) biomarkers can distinguish AD comorbidity in LBD patients.

**Methods:**

We examined plasma Aβ_40_, and Aβ_42_ in 15 patients with Aβ negative LBD, 10 with Aβ positive LBD, and 37 with AD. In addition, patients underwent amyloid‐positron emission tomography imaging (^11^C Pittsburgh compound‐B) or cerebrospinal fluid Aβ_42_ test.

**Results:**

The plasma Aβ_40_/Aβ_42_ ratio was significantly decreased in patients with Aβ+ LBD compared with those with Aβ‐ LBD (p = 0.037) and could accurately classify the two diseases (area under the curve, 0.727).

**Conclusions:**

Plasma Aβ_40_/Aβ_42_ ratio might be a useful marker for comorbid AD pathology in LBD.